# Molecular markers to characterize the hermaphroditic reproductive system of the planarian *Schmidtea mediterranea*

**DOI:** 10.1186/1471-213X-11-69

**Published:** 2011-11-10

**Authors:** Tracy Chong, Joel M Stary, Yuying Wang, Phillip A Newmark

**Affiliations:** 1 Department of Cell and Developmental Biology, University of Illinois at Urbana-Champaign, 601 South Goodwin Avenue, Urbana, Illinois 61801, USA; 2 Neuroscience Program, University of Illinois at Urbana-Champaign, 505 South Goodwin Avenue, Urbana, Illinois 61801, USA; 3 Howard Hughes Medical Institute, University of Illinois at Urbana-Champaign, 601 South Goodwin Avenue, Urbana, Illinois 61801, USA; 4 Department of Pediatrics, University of Colorado School of Medicine, Aurora, Colorado 80045, USA

## Abstract

**Background:**

The freshwater planarian *Schmidtea mediterranea *exhibits two distinct reproductive modes. Individuals of the sexual strain are cross-fertilizing hermaphrodites with reproductive organs that develop post-embryonically. By contrast, individuals of the asexual strain reproduce exclusively by transverse fission and fail to develop reproductive organs. These different reproductive strains are associated with distinct karyotypes, making *S. mediterranea *a useful model for studying germline development and sexual differentiation.

**Results:**

To identify genes expressed differentially between these strains, we performed microarray analyses and identified >800 genes that were upregulated in the sexual planarian. From these, we characterized 24 genes by fluorescent *in situ *hybridization (FISH), revealing their expression in male germ cells or accessory reproductive organs. To identify additional markers of the planarian reproductive system, we also used immuno- and fluorescent lectin staining, identifying several antibodies and lectins that labeled structures associated with reproductive organs.

**Conclusions:**

Collectively, these cell-type specific markers will enable future efforts to characterize genes that are important for reproductive development in the planarian.

## Background

While the planarian has re-emerged as an animal model for studying regeneration and stem cell biology [1-5], recent studies of *Schmidtea mediterranea *have also contributed to understanding germ cell biology and sexual development [6-9]. Two modes of specifying germ cells have evolved among animals, involving either localized determinants or inductive signaling [10-12]. Sexual planarians have a reproductive system that can be regenerated *de novo *from stem cells after amputation [9,13], suggesting that they specify their germline inductively like mammals and many basal metazoans [10,14,15].

**Figure 1:**
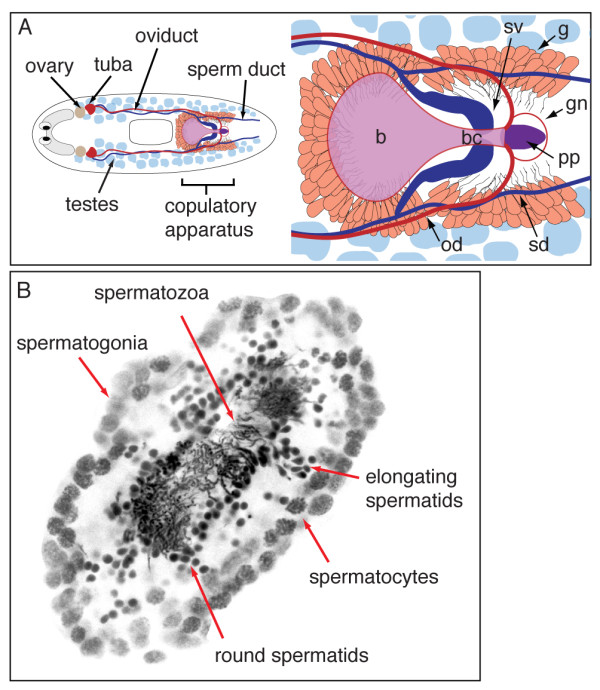
**The planarian reproductive system**. (A) Diagram depicting the generalized reproductive system in sexual *S. mediterranea*. Right, enlarged view of the copulatory apparatus. Bursa (b); bursal canal (bc); seminal vesicles (sv); sperm ducts (sd); oviducts (od); glands (g); gonopore (gn); penis papilla (pp). (B) Schematized view of different germ cell types in the planarian testes.

*S. mediterranea *is a powerful model for understanding germ cell development due, in part, to the existence of distinct sexual and asexual strains that allow identification and characterization of genes involved in the different modes of reproduction. The reproductive system of the hermaphrodite consists of the male and female gonads, as well as accessory reproductive organs [7]. Numerous testes are distributed dorsolaterally along the animal, and a pair of ovaries is situated more ventrally at the posterior region of the cephalic ganglia (brain). Ciliated oviducts and sperm ducts running along the nerve cords lead to the copulatory apparatus, comprised of the gonopore (gn), seminal vesicles (sv), copulatory bursa (b), bursal canal (bc), penis papilla (pp) and various glands (g) (Figure 1A) [7]. In each planarian testis lobe, spermatogenesis proceeds from the periphery to the lumen. Spermatogonia undergo three mitotic divisions with incomplete cytokinesis to produce eight primary spermatocytes that enter meiosis, differentiate into 32 spermatids, and mature into spermatozoa (Figure 1B) [16]. The sperm are released into the sperm ducts that funnel sperm to the seminal vesicles. When *S. mediterranea *mate, sperm from one animal is transferred to its partner and deposited via the bursal canal into the copulatory bursa. The sperm then travel back down the bursal canal into the oviducts and collect in the tuba, an enlarged portion of the oviducts just outside the ovaries (Figure 1A) [17,18]. As mature oocytes leave the ovary, they are fertilized by sperm stored in the tuba. The fertilized eggs then make their way down the oviduct, and yolk cells are added to the outside of the egg by the vitelline (yolk) glands that line the oviduct. Several embryos and yolk cells are packaged into a single egg capsule. The glands around the genital atrium are involved in the synthesis and deposition of egg capsules [18,19].

In contrast to the sexual strain, the asexual strain of *S. mediterranea *does not develop reproductive organs, and reproduces exclusively by transverse fission. While asexual planarians do not elaborate well-developed gonads, they do possess primordial germ cells that fail to differentiate further [8,9,20,21]. Although the asexual strain is distinguished by a chromosomal translocation, the exact mechanisms that account for the two divergent modes of reproduction are unknown [22,23]. However, neuropeptide signaling was recently shown to be required for the development of reproductive organs in sexual planarians. RNA interference (RNAi) knockdown of a single neuropeptide gene, *npy-8 *[GenBank: BK007010], resulted in animals that failed to develop or maintain reproductive organs. Interestingly, *npy-8 *mRNA was not detected in asexual planarians [6].

The existence of two divergent modes of reproduction in a single species presents a unique opportunity to identify conserved and species-specific genes that are important for germ cell development and reproductive maturation. Thus, we used two approaches to characterize differences between these strains: (i) microarray analysis to identify genes that are expressed differentially between sexual and asexual planarians, followed by *in situ *hybridization to identify the cell types in which these genes are expressed; and (ii) morphological analysis using confocal microscopy to identify cell type-specific markers that label components of the sexual reproductive system. We show that several genes identified through this transcriptional comparison serve as useful markers for somatic and germ cells of the planarian reproductive system. We also introduce several antibodies and fluorescent lectin-conjugates that will be useful for visualizing components of the planarian reproductive system. These studies provide complementary approaches for studying the genetic and morphological differences between sexual and asexual modes of reproduction in planarians.

## Results and Discussion

### Identification of genes with significantly higher expression in sexual planarians

**Figure 2:**
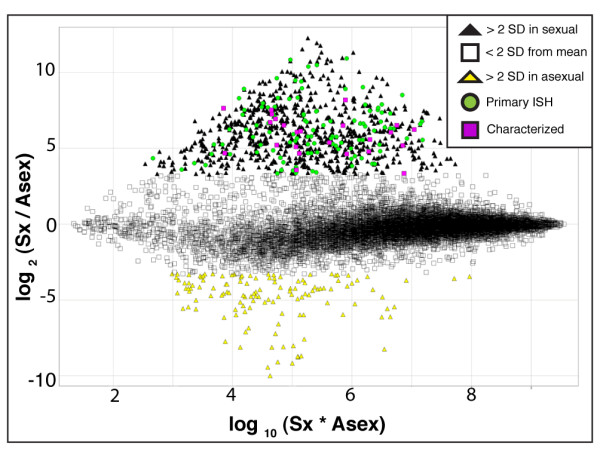
**Transcriptional analysis of sexual versus asexual planarians**. Ratio-intensity (R-I) scatter plot depicting the results of oligonucleotide microarray comparing gene expression between mature sexual planarians (Sx) and asexual (Asex) planarians. Standard deviation (SD); in situ hybridization (ISH); transcripts that showed upregulation (≥ 2 SD) in sexuals (black triangles); transcripts that showed upregulation (≥ 2 SD) in asexuals (yellow triangles); transcripts from primary in situ hybridization screen that were expressed in the planarian reproductive system (green circles); transcripts characterized in this study (purple squares); transcripts showing changes ≤ 2 SD (white squares).

To compare gene expression profiles of sexual and asexual planarians and to identify genes involved in sexual development we performed microarray analysis using custom oligonucleotide arrays with probes representing 16,786 unique *S. mediterranea *transcripts. Analysis of transcriptional profiles from sexual and asexual planarians revealed 951 genes with differential expression greater than 2 standard deviations (approximately 9.7-fold difference) from the mean. We applied this stringent cut-off to enrich for the most differentially expressed genes that would potentially serve as markers of mature reproductive tissues. Of these differentially expressed genes, 822 were expressed at higher levels in sexual animals and 129 were expressed at higher levels in asexual animals (Figure 2). To assign putative functions to genes upregulated in sexual animals, we searched for conserved domains and performed Cluster of Orthologous Group (COG) analyses [24]. For the genes upregulated in sexual animals, these analyses identified 346 of 822 (42%) genes with putative roles in diverse processes; the remaining 476 genes were novel, with no obvious conserved domains. Of the genes with conserved functions, the greatest number could be assigned to the functional categories cytoskeleton, signal transduction, and cell cycle control/cell division/chromosome partitioning. Additionally, our analyses found upregulated genes that shared homology to proteins of unknown function in other organisms (Additional file 1, Table S1). Functional studies of these conserved genes in planarians should provide insight into the roles of these genes in reproductive processes. For the genes upregulated in asexual animals, putative functional categories were assigned to only 14 of 129 genes (11%) using COG analyses (Additional file 2, Table S2); the remaining 115 genes (89%) were novel. Further examination of these genes should shed light on the mechanisms of asexual reproduction in planarians.

### Genes that are upregulated in sexual planarians are expressed in the planarian reproductive system

To validate our microarray results, a primary whole-mount *in situ *hybridization (WISH) screen involving 122 genes that showed significant differential expression in the sexual planarians (upregulated ≥ 2 standard deviations from the mean) was performed on mature sexual animals. We focused on genes from four different COG functional categories: signal transduction, transcription, cytoskeleton, and genes of unknown function. Of the 122 genes examined, 100 (82%) were expressed in the reproductive system: 96 genes were expressed in the testes, and 4 genes were expressed in accessory reproductive organs (data not shown). To characterize further the cellular distribution of several of the transcripts, 24 that were expressed either in accessory reproductive organs or during different stages of spermatogenesis were analyzed using fluorescence *in situ *hybridization (FISH) (Additional file 3, Figure S1 and Additional file 4, Table S3).

**Figure 3:**
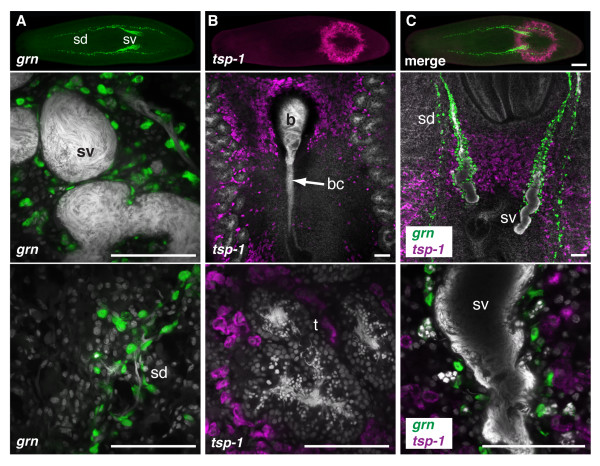
***grn *and *tsp-1 *are expressed in accessory reproductive organs**. (A-C, top panel) Whole mount view of two-color FISH showing expression of *grn *(green) and *tsp-1 *(magenta) in sexual *S. mediterranea*. Anterior of animal is to the left. (A-C, middle and bottom panels) Single confocal optical sections showing expression of *grn *(green) and/or *tsp-1 *(magenta). Nuclei are counterstained with DAPI (grey). (A) *grn *is expressed in the seminal vesicles and sperm ducts. (B) *tsp-1 *is expressed in glands around the copulatory bursa and bursal canal. The *tsp-1*-expressing cells are interspersed between testis lobules near the copulatory apparatus. (C) Two-color FISH showing distinct populations of cells expressing either *grn *or *tsp-1*. Sperm duct (sd); seminal vesicles (sv); bursa (b); bursal canal (bc); testes (t). Scale bars: (A-C, top panel) 500 μm; (A-C, middle and bottom panels) 100 μm.

Transcripts of two genes were detected in accessory reproductive organs. A gene similar to *granulin *(*Smed-grn *[GenBank: DN304193.1]; for brevity, we will drop the prefix *"Smed" *from the remaining genes described here) was expressed in a subset of cells forming the sperm ducts and seminal vesicles, components of the "male" reproductive system (Figure 3A and Table 1). The second gene, sharing homology with tetraspanin genes (*tsp-1 *[GenBank: DN305069.1]), was expressed in glands around the atrium, a "female" structure often associated with egg capsule production. The glands span from the dorsal to the ventral surface of the animal and are interspersed between the testis lobules close to the copulatory apparatus (Figure 3B and Table 1). Simultaneous labeling by two-color FISH showed that *grn *and *tsp-1 *were expressed in distinct populations of cells, confirming that some genes are expressed specifically in either male or female reproductive structures (Figure 3C).

**Table 1:** Genes identified through the sexual/asexual array are expressed in accessory reproductive organs or enriched in different stages of germ cell development in the testes.

Gene	Accessory reproductive organs		Testes		
	
	Sperm duct/seminal vesicles	Glands around copulatory apparatus	Spermatogonia	Spermatocytes	Spermatids
*grn*	+				

*tsp-1*		+			

*msy4*			+	+	+

*thmg-1*			+	+	+

*pde*			+	+	+

*plastin*			+	+	+

*tkn-2*			+	+	+

*tkn-1*				+	

*tplh*				+	+

*pp2*					+

*pka*					+

**Figure 4:**
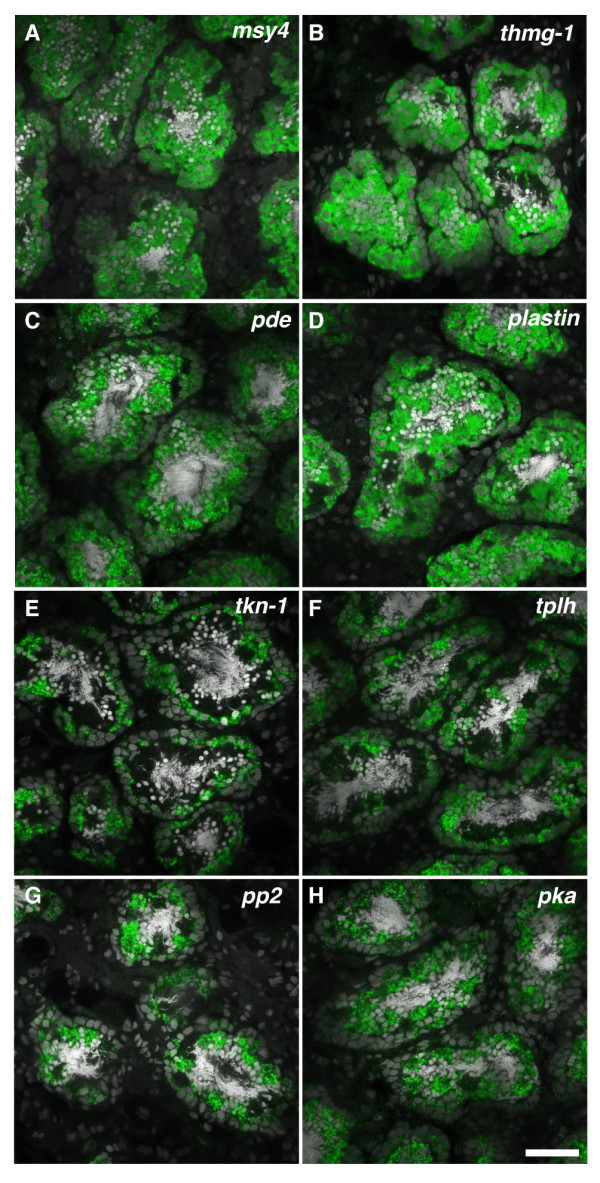
**Genes upregulated in the sexual planarian that are expressed in the testes**. (A-H) Single confocal sections showing FISH for genes (green) expressed in the testes. Nuclei are counterstained with DAPI (grey). (A-D) *msy4, thmg-1, pde*, and *plastin *transcripts are expressed in all germ cell types in the testes except mature sperm. (E) *tkn-1 *transcripts are enriched in spermatocytes. (F) *tplh *transcripts are enriched in spermatocytes and spermatids. (G and H) *pp2 *and *pka *transcripts are enriched in spermatids. Scale bar: 40 μm.

We also used FISH to determine the cell types in which testes-specific genes were expressed (Table 1). This analysis found transcripts expressed broadly throughout the testes; for example, transcripts for four genes (*msy4 *[GenBank: BK007100], *thmg-1 *[GenBank: DN307831.1], *pde *[GenBank: HO008078.1, DN313728.1], and *plastin *[GenBank: HO007660.1, DN311193.1]) were detected in all male germ cell stages except fully mature spermatozoa (Figure 4A-D). MSY4, a Y-box containing protein is expressed in the male germ cells of mice [25][GenBank: AF246224.1] and the planarian *Dugesia etrusca *[26][GenBank: AJ439094.1]; knockdown of *msy4 *has been shown to disrupt spermatid elongation in *S. mediterranea *[8]. This result validates this approach for identifying genes expressed in planarian germ cells. Furthermore, some transcripts were more abundantly expressed in different cell populations, such as spermatocytes (*tkn-1 *[GenBank: HO007229.1, DN309082.1], Figure 4E), in spermatocytes and spermatids (*tplh *[GenBank: HO005509.1, DN311232.1], Figure 4F), or in more differentiated germ cells in the luminal region that likely correspond to spermatids (*pp2 *[GenBank: HO006984.1, DN315773.1], Figure 4G and pk*a *[GenBank: HO007035.1, DN316100.1], Figure 4H).

**Figure 5:**
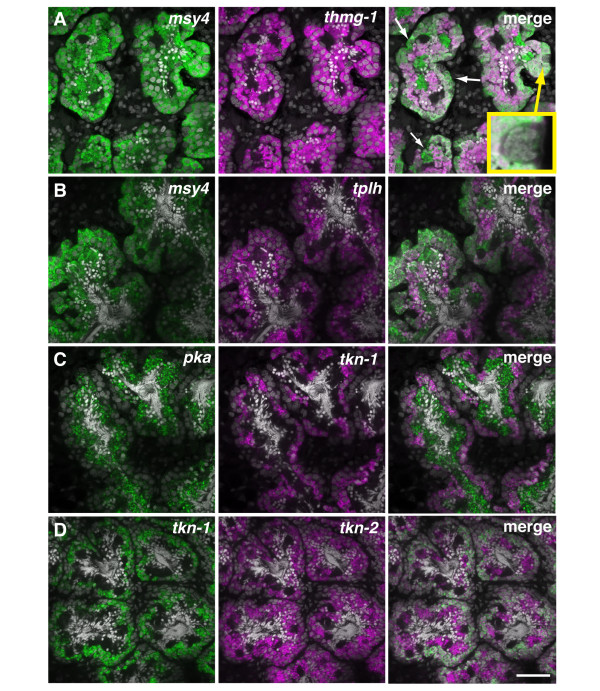
**Differential gene expression in distinct populations of male germ cells in the testes**. (A-D) Single confocal optical sections showing double FISH of genes (magenta/green) expressed in different populations of male germ cells in the testes. Nuclei are counterstained with DAPI (grey). A) *msy4 *and *thmg-1 *are broadly expressed in differentiating male germ cells. Arrows indicate cells that lack *thmg-1 *expression. Cells lacking both *msy4 *and *thmg-1 *(inset) are likely to be somatic cells associated with the testes. B) *msy4 *transcripts are detected in all germ cell stages, *tplh *transcripts are enriched in spermatocytes and spermatids. C) *pka *transcripts are upregulated in spermatids, *tkn-1 *transcripts are detected in spermatocytes. D) Tektin paralogs, *tkn-1 *and *tkn-2*, are expressed in distinct stages of germ cell differentiation. Scale bar: 40 μm.

To confirm that these genes were enriched in distinct germ cell populations within the testes, we performed two-color FISH. Although transcripts of *msy4 *and *thmg-1 *are broadly expressed in differentiating male germ cells, it appeared that *msy4 *transcripts were enriched in subpopulations of cells lacking *thmg-1 *expression (Figure 5A, white arrows). We also observed cells located in the outer-most layer of the testes that were lacking both *msy4 *and *thmg-1 *expression, suggesting that these cells could be the somatic cells of the testes (Figure 5A, boxed inset). While *msy4 *transcripts were detected in all male germ cell stages, *tplh *transcripts were enriched in spermatocytes and spermatids (Figure 5B). The spermatocytes expressing *tkn-1 *transcripts can be distinguished from the more differentiated germ cells that showed *pka *upregulation (Figure 5C). In contrast to expression of *tkn-1 *mRNA in the spermatocytes, *tkn-2 *[GenBank: HO008093.1, DN313931.1] was more broadly expressed in the different germ cell types in the testis (Figure 5D). Tektins are a conserved component of cilia and flagella [27], and our observations indicate that these two *tektin *paralogs (*tkn-1 *and *2*) are expressed in distinct stages of male germ cell differentiation.

Mechanisms regulating different stages of germ cell differentiation are vital for the production of gametes. Based on the assigned putative functions of the genes examined and the enrichment of their mRNA transcripts in different domains of the testes, these genes may be involved in regulating the differentiation of male germ cells. Several studies have shown that germ cells rely upon post-transcriptional regulation to maintain their genomic plasticity [12,28]. A recent functional genomic screen in *S. mediterranea *showed that RNA-binding proteins act as critical regulators of germ cell development in planarians [8], similar to germ cell regulation across metazoans, in which RNA-binding proteins regulate germ cell proliferation, stem cell maintenance, and sex determination [29-32]. For future work on genes upregulated in sexual planarians, it will be important to functionally characterize genes from other COG categories, such as RNA processing and modification, as well as translation, ribosomal structure and biogenesis, with emphasis on genes encoding RNA-binding proteins.

The aforementioned screen to identify genes required for germ cell development in planarians [8] used microarrays to investigate changes in gene expression in animals lacking germ cells after *nanos *[GenBank: EF035555] RNAi. These experiments were designed to identify differences in the "early" germ cell populations found in asexual planarians and juvenile sexual planarians (i.e., well before reproductive maturity); however, they would not detect genes expressed in the latest stages of reproductive maturation. By contrast, the experiments reported here to compare asexual and fully mature sexual planarians should detect genes expressed in the accessory reproductive organs and/or during the latest stages of reproductive system development. The *nanos *knockdown experiments identified 103 genes that were downregulated after *nanos *(RNAi) in asexual planarians and 275 genes that were downregulated after *nanos *(RNAi) in juvenile sexual planarians [8]; whereas, we found >800 genes that were dramatically upregulated in mature sexual planarians relative to asexuals. Given the large number of reproductive organs (gonads and the many accessory organs) that are present in mature sexual animals, it is not surprising that there are many more genes expressed differentially and at much greater differential levels than were observed in the *nanos *RNAi experiments.

Comparing these data sets revealed that 237/275 genes (86%) that were downregulated in juvenile sexual *nanos *(RNAi) animals (i.e., lacking germ cells) were upregulated (>2 s.d.) in sexual planarians relative to asexuals. Similarly, 88/103 genes (85%) that were downregulated in asexual *nanos *(RNAi) animals were upregulated (>2 s.d.) in sexual planarians. Of the 13 genes shown to be required for different stages of germ cell development [8] nine were upregulated >2 s.d. and two were upregulated >1 s.d. (>3.12-fold) in sexual planarians. Thus, there was excellent agreement between these different data sets. Because asexual planarians also possess early *nanos*-expresssing germ cells, it is possible that some of the genes that do not show differential expression between asexual and sexual planarians are expressed in early germ cells. Among the genes characterized here by *in situ *hybridization, 15/24, including those expressed in the somatic accessory structures were not expressed differentially in *nanos *(RNAi) versus control sexuals, validating this complementary approach to identify genes involved in planarian sexual development.

Our *in situ *hybridization analyses did not detect genes whose transcripts were expressed exclusively in the ovaries. The difficulty of isolating ovary-specific genes is most likely based upon the paucity of ovarian tissue relative to the animal as a whole. The two ovaries reside at the base of the cephalic ganglia, whereas the testes are distributed throughout the dorso-lateral margin, along most of the length of the animal. Several other accessory reproductive organs also occupy a much greater portion of the planarian body than do the ovaries. Because these experiments have used RNAs isolated from whole planarians, ovarian RNAs are likely to be under-represented relative to testis-enriched RNAs. Thus, isolation of additional ovary-specific transcripts will require other experimental approaches.

### Immunofluorescent and fluorescent lectin-conjugate labeling of the planarian reproductive system

The majority of studies describing the planarian reproductive system have been based on histological sections and electron microscopy. While these studies have been very important in describing the morphology of the reproductive organs, they often require serial sectioning and reconstruction, both of which are technically challenging and time consuming. To characterize the reproductive anatomy in whole-mount sexual planarians, we tested several reagents that could potentially label various components of planarian reproductive organs (Table 2). In addition to commercially available antibodies, a rabbit pre-immune serum that reacts with epitopes in planarian muscle cells (anti-muscle) was also used [33]. Lectins, carbohydrate-binding proteins [34,35], have been used to label cells or tissues- primarily the secretory cells, their cytoplasmic projections, and terminal pores in asexual planarians [36]. Here, we report four fluorescent lectin-conjugates that label reproductive structures in the sexual planarian.

**Table 2:** Antibodies and lectins that label accessory reproductive organs and gonads in *S. mediterranea*.

Antibody	Accessory reproductive organs						Testes		
	
	Oviduct/tuba	Bursa canal	Glands around the copulatory apparatus	Seminal vesicles	Sperm ducts	Penis papilla	Sperm flagella	Membranes in/around testes	Spermatogonia/spermatocytes/spermatids
Tubulin δ2*	+				+		+		

Phosphotyrosine^^^	+	+		+		+			

Muscle (pre-immune serum)^^^	+	+		+					

Carbonic anhydrase^^^	+								

**Lectin**									

Peanut agglutinin (PNA)*		+	+						+

Ricinus communis agglutinin (RCA)*			+						

Erythrina cristagalli lectin (ECL)*			+						

Lens culinaris agglutinin (LCA)*								+	

^ Animals were fixed with methacarn.

* Animals were fixed with formaldehyde.

**Figure 6:**
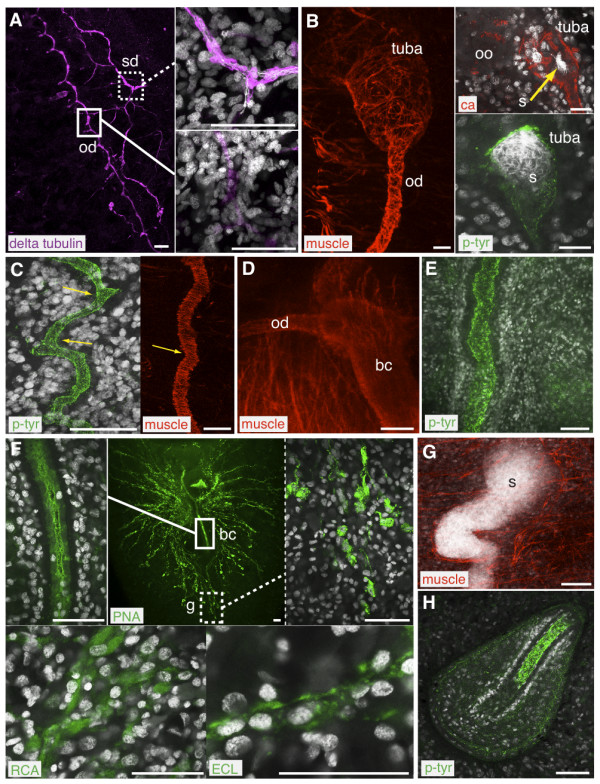
**Immunofluorescent and lectin labeling of the planarian reproductive system**. (A-H) Confocal images of immuno or lectin staining in red/magenta/green, DAPI in grey. A) Oviducts (od) and sperm ducts (sd) are labeled by anti-tubulin δ2 (delta tubulin). B) The tuba and oviducts are labeled by anti-muscle (muscle), anti-phosphotyrosine (p-tyr) and anti-carbonic anhydrase (ca). Oocytes (oo) are found in the ovary anterior to the tuba, sperm (s) collect in the tuba. C) Bends in the oviducts (yellow arrows). D) The bursal canal (bc) connects to the oviducts. E) The bursal canal is also visualized by anti-phosphotyrosine. F) Lectin Peanut agglutinin (PNA) labels the bursal canal and glands around the copulatory apparatus, lectin *Ricinus communis *agglutinin (RCA) and *Erythrina cristagalli *lectin (ECL) also label glands around the compulatory apparatus. G) Seminal vesicles containing sperm are suspended in a network of muscle fibers. H) The penis papilla is labeled by anti-phosphotyrosine. Scale bars: (A, C-H) 50 μm; (B) 20 μm.

The oviducts and tuba, accessory reproductive organs that are ciliated and ensheathed by muscle fibers, were labeled by anti-tubulin δ2 (Figure 6A, solid box), anti-muscle, and anti-carbonic anhydrase (Figure 6B). A monoclonal antibody that recognizes phosphorylated tyrosine residues (anti-phosphotyrosine) is a general marker that labels multiple structures in the planarian [37]; this antibody also enables visualization of the oviducts and tuba (Figure 6B). We observed that the oviducts were not smooth, straight tubes; rather, they often bent (Figure 6C, arrows) and occasionally looped (Additional file 5, Figure S2A, left panel). Preliminary experiments to label animals of different sizes and maturity have shown that the oviducts develop as individual fragments that join with one another to form a continuous tube, and that the bends in the tube are sites where the individual fragments meet (Additional file5, Figure S2A, right panel). The oviducts connect to the bursal canal, which also labeled with the anti-muscle (Figure 6D) and anti-phosphotyrosine (Figure 6E) antibodies, as well as the lectin Peanut agglutinin (PNA, Figure 6F, solid box). The glands around the copulatory apparatus were recognized by three lectins: PNA (Figure 6F, dashed box), *Erythrina cristagalli *lectin (ECL, Figure 6F, bottom right) and *Ricinus communis *agglutinin (RCA, Figure 6F, bottom left).

The male accessory reproductive organs were also labeled by a variety of antibodies and lectins. The ciliated sperm ducts were labeled by anti-tubulin δ2 (Figure 6A, dashed box) while the seminal vesicles, tubular structures suspended in a network of muscle fibers, were visualized with anti-muscle (Figure 6G) and anti-phosphotryosine immunostaining (Additional file 5, Figure S2B). The penis papilla was labeled by anti-phosphotyrosine (Figure 6H) and ECL (Additional file 5, Figure S2C). Finally, the common gonopore could be visualized by staining with muscle antibody (Additional file 5, Figure S2D).

**Figure 7:**
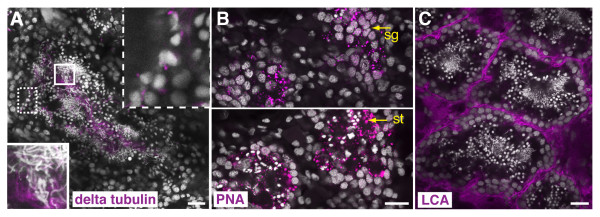
**Immunofluorescent and lectin labeling of the planarian testes**. (A-C) Single confocal optical sections showing either immuno or lectin staining (magenta). Nuclei are counterstained with DAPI (grey). (A) Antibody labeling with anti-tubulin δ2 (delta tubulin). Insets show magnified views of flagella (magenta) on round spermatids (dashed box) and sperm (solid box). (B) Peanut agglutinin (PNA) staining in the testes. Top panel shows a confocal section of a testis with labeling of spermatogonial cells (sg), bottom panel shows a more ventral confocal section of the same testis with labeling of spermatids (st). (C) *Lens culinaris *agglutinin (LCA) labels the membranes in and around the testes. Scale bars: 20 μm.

In addition to accessory reproductive organs, we also used immuno and lectin staining to label testes. The antibody specific for tubulin δ2 selectively labeled microtubules in the flagella of sperm, allowing visualization of haploid male germ cells in the process of spermiogenesis; including the growing flagella at the round spermatid stage (Figure 7A, dashed box) and the elongation of spermatids into mature sperm (Figure 7A, solid box). Using this antibody, it was also possible to visualize the flagella of the mature sperm as they travel along the oviducts, bursa and bursal canal (Additional file 5, Figure S2E). Intriguingly, the sperm seem to be clustered with their flagella oriented towards the periphery. Further examination of the sperm as they traverse the ducts will provide clues on how the sperm are transported through the reproductive system. In the testes, lectin PNA staining was distributed in a punctate pattern on the surface of pre-meiotic and post-meiotic germ cells (Figure 7B), while the lectin *Lens culinaris *agglutinin (LCA) labeled cell membranes in and around the testes (Figure 7C).

The markers described thus far allow the labeling of male and female reproductive structures of the planarian. An interesting question in simultaneous hermaphrodites is whether male and female reproductive structures are co-dependent on each other. While some genes function in both male and female developmental pathways, there are two potentially distinct pathways that are responsible for either male or female sexual development. This idea is supported by the fact that it is possible to use RNAi to generate animals devoid of male germ cells [8]. The presence of sex-specific pathways in planarians like *S. mediterranea *could allow the evolution of simultaneous hermaphrodites into distinct male or female sexes, as seen in more derived flatworm species like the parasitic schistosomes. Interestingly, glands that are labeled with lectins in planarians are also labeled by similar lectins in *Schistosoma mansoni *females, suggesting a common evolutionary origin for these organs [38]. The ability to visualize both male and female components of the planarian reproductive system with antibodies, lectins, and FISH as shown here will prove useful as we proceed with functional studies to identify genes involved in sexual development to gain insight into the mechanisms responsible for sex-specific development.

## Conclusions

Using microarray analyses, we have identified >800 genes that are upregulated in the sexual vs. asexual planarian. A subset of these genes, validated through whole-mount *in situ *hybridization, provides markers of the planarian reproductive system. Their differential expression in differentiating germ cells will be useful for dissecting the spatial and temporal development of planarian reproductive organs. These transcriptomic data, combined with the ability to simultaneously label both male and female components of the planarian reproductive system through the markers identified in this study, will enable functional studies to dissect pathways and mechanisms that are involved in inductive germ cell specification, as well as sexual differentiation and development.

## Methods

### Planarian culture

Sexual and asexual *S. mediterranea *were maintained as previously described [9,37], and starved at least 1 week before use. For all *in situ *hydridization, immuno and lectin experiments, sexually mature animals were used.

### Sequences for oligonucleotide arrays

Sequences from three *S. mediterranea *EST libraries (Alvarado et al. 2002, Zayas et al. 2005, unpublished ESTs in NCBI) were obtained from NCBI and assembled into 17,568 unique sequences using a variety of approaches (e.g. BLAST, Sequencher and CAP3, and hand curation). These sequence data were used to generate an oligonucleotide array that was submitted to Roche Nimblegen for probe creation. Of the 17,568 sequences submitted, 628 had no suitable regions for probe creation; 129 shared all their probes with 1 other sequence; 12 shared all their probes with 2+ other sequences; and 357 had suitable sequence available for less than 10 probes. After these considerations, the oligonucleotide array had probes representing 16,786 ESTs. Each gene was represented by 10 probe pairs with a mean length of 60 nucleotides.

### RNA extraction and purification

Total RNA was extracted from equivalent weights of asexual and sexual animals using a modified Trizol protocol that included an optional spin after homogenization and isopropanol/high salt solution precipitation (Invitrogen). RNA was treated with RNase-free DNAse (Promega) using standard protocols, purified using an RNeasy Mini Spin kit (Qiagen), and quantified with both a Nanodrop ND-1000 spectrophotometer (Thermo Scientific) and an Agilent 2100 Bioanalyzer (Agilent Technologies). The purified RNA samples were sent to Roche Nimblegen for labeling and hybridization to custom oligonucleotide arrays.

### Oligonucleotide array data analysis

Expression data were received from Roche Nimblegen Inc. and EST calls were generated using the Robust Multichip Average (RMA) algorithm [39,40]. Z-scores were calculated to assess differential expression. Microarray data have been deposited in the Gene Expression Omnibus (Accession number GSE32450).

### Riboprobe synthesis and whole-mount in situ hybridization

To generate riboprobes for *in situ *hybridization, clones from the hermaphroditic *S. mediterranea *EST Database [41] were used as templates. In vitro transcription reactions with T3 RNA polymerase were performed using standard approaches allowing the incorporation of digoxigenin-12-UTP (Roche), fluorescein-12-UTP (Roche), or dinitrophenol-11-UTP (PerkinElmer). Whole-mount *in situ *hybridization of mature sexual planarians was performed as previously described [8,42]. *In situ *hybridizations were conducted either manually or with the Insitu ProVS (Intavis AG Bioanalytical Instruments). For FISH, following post-hybridization washes and blocking, animals were incubated in anti-digoxigenin peroxidase, (1:1000 [Roche]), anti-FITC peroxidase, (1:1000 [Roche]) and/or anti-dinitrophenol peroxidase, (1:100 [PerkinElmer]) overnight at 4°C. Samples were washed at least 6 times (20 min each), and developed with FITC- or Cy3-Tyramide (PerkinElmer) using the manufacturer's protocol. For double FISH, the peroxidase after the first tyramide development was quenched with 2% H_2_O_2 _in PBTX (1× PBS + 0.1% Triton X-100) for 1 hour before subsequent antibody incubation. Samples were mounted in Vectashield (Vector Laboratories) for imaging.

### Whole-mount immunofluorescence and Fluorescent Lectin-Conjugate Staining

Planarians were killed with 2% HCl for 5 minutes on ice and fixed for 2 hours with either methacarn (methanol: chloroform: acetic acid [6:3:1]), or 4% formaldehyde in 1× PBS. Following formaldehyde fixation, animals were bleached in 6% H_2_O_2 _in 1× PBS for one or two nights depending on size/pigmentation. For methacarn fixation, animals were incubated in methanol (MeOH) for 1-2 hours before bleaching in 6% H_2_O_2 _in MeOH and then rehydrated in a 75, 50, 25% MeOH/PBTX (1× PBS + 0.3% Triton X-100) gradient. For both methacarn and formaldehyde fixed samples, animals were washed twice (five minutes each) with PBTX, and incubated in blocking buffer (0.6% IgG free BSA, 0.45% fish gelatin in PBTX) for 2-4 hours. Primary antibody incubation was performed overnight at 4°C at the following concentrations (dilutions in blocking buffer): carbonic anhydrase II human erythrocytes rabbit polyclonal antibody (1:100 [Chemicon International, AB1828]), phospho-tyrosine mouse monoclonal antibody (1:500 [Cell Signaling Technology, 9411]), anti-tubulin δ2 rabbit polyclonal antibody (1:200 [Millipore, AB3203]), rabbit pre-immune serum/anti-muscle (1:500 [generated by Francesc Cebrià and Tingxia Guo, [33]]). After at least six one-hour washes in PBTX, animals were incubated in secondary antibody (goat anti-rabbit Alexa 568, 1:1000 [Molecular Probes, Invitrogen, A11036]; goat anti-mouse Alexa 488, 1:400 [Molecular Probes, Invitrogen, A11029]) overnight at 4°C. For staining with lectins, samples were incubated overnight at 4°C with FITC- or rhodamine-conjugated lectins (1:500 [Vector Laboratories]) diluted from 2 mg/ml stocks in blocking buffer. All immuno and lectin samples were counterstained with DAPI. Animals were washed several times before they were mounted in Vectashield and flattened for imaging.

### Microscopy

Samples were imaged with a Leica M205A stereomicroscope and DFC420 camera, a Zeiss Stereo Lumar V12 and/or a LSM 710 confocal microscope. Confocal microscopy was performed as previously described [6]. Images were processed using either Zen 2008/9 (Carl Zeiss) or Adobe Photoshop CS4 (Adobe).

## Authors' contributions

TC carried out the *in situ *hybridizations, lectin and immuno-stainings, participated in the analysis of upregulated microarray transcripts, and drafted the manuscript. JMS designed the microarray, performed the microarray analysis and wrote the corresponding section of the manuscript. YW participated in the microarray analysis. PAN conceived of the study and helped with the experimental design and manuscript writing. All authors read and approved the final manuscript.

## Supplementary Material

Additional file 1 **Table S1 - Clusters of Orthologous Groups (COG) functional categories for genes upregulated in sexual planarians**. Genes were assigned putative functions based on their conserved domains. Some genes are assigned more than one functional category. Click here for file

Additional file 2 **Table S2 - Clusters of Orthologous Groups (COG) functional categories for genes upregulated in asexual planarians**. Genes were assigned putative functions based on their conserved domains. Some genes are assigned more than one functional category. Click here for file

Additional file 3 **Figure S1 - Genes upregulated in the sexual planarian that are expressed in the testes**. Right panels are fluorescent in situ hybridizations (green), nuclei are counterstained with DAPI (magenta). Left panels are whole mount in situ hybridizations developed with nitro blue tetrazolium/5-bromo-4-chloro-3-inodlyl phosphate (NBT/BCIP). Scale bars: 40 μm. Click here for file

Additional file 4 **Table S3 - Genes upregulated in the sexual planarian that are expressed in the testes and accessory reproductive organs of the planarian**. Click here for file

Additional file 5 **Figure S2 - Immunofluorescent and lectin labeling of the planarian reproductive system**. (A, D, E) Maximum projection of a confocal z-stack. (B, C) Single confocal optical sections. (A, B, D, E) Immunostaining in red/magenta/green, DAPI in grey. (C) Lectin staining in green, DAPI in grey. (A) Looped section of the oviduct, labeled by anti-muscle. Arrows in right image indicate individual oviduct fragments in a sexually immature animal, labeled by anti-muscle. (B) Antibodies against phosphotyrosine (p-tyr) label the seminal vesicles. (C) *Erythrina cristagalli *lectin (ECL) labels the penis papilla. (D) Muscle fibers of the gonopore are labeled with anti-muscle. (E) Flagella of the sperm are labeled with anti-tubulin δ2 (delta tubulin). Left image shows sperm in the bursa (b) and bursal canal (bc). Right image shows sperm in the oviducts (od). Scale bars: (A, B, D, E) 50 μm; (C) 20 μm. Click here for file
