# Supplementary material for: Molecular markers to characterize the hermaphroditic reproductive system of the planarian Schmidtea mediterranea
Source: BMC Dev Biol. 2011 Nov 10;11:69. doi: 10.1186/1471-213X-11-69 (PMC3224759; doi:10.1186/1471-213X-11-69)
Supplement: Additional file 1 — Table S1 - Clusters of Orthologous Groups (COG) functional categories for genes upregulated in sexual planarians. Genes were assigned putative functions based on their conserved domains. Some genes are assigned more than one functional category. [file 1471-213X-11-69-S1.DOC]

| **COG Functional Category** | **Percentage (out of 346 genes)** | **Number of genes** |
| --- | --- | --- |
| Cytoskeleton | 21.4 % | 74 |
| Signal transduction mechanisms | 20.0 % | 69 |
| Cell cycle control, cell division, chromosome partitioning | 18.5 % | 64 |
| Function unknown | 14.7 % | 51 |
| Posttranslational modification, protein turnover, chaperones | 9.3 % | 32 |
| Inorganic ion transport and metabolism | 8.1 % | 28 |
| Carbohydrate transport and metabolism | 7.8 % | 27 |
| Transcription | 6.1 % | 21 |
| Amino acid transport and metabolism | 4.9 % | 17 |
| Defense mechanisms | 4.9 % | 17 |
| Energy production and conversion | 4.3 % | 15 |
| RNA processing and modification | 4.1 % | 14 |
| Intracellular trafficking, secretion, and vesicular transport | 3.8 % | 13 |
| Cell wall/membrane/envelope biogenesis | 2.0 % | 7 |
| Lipid transport and metabolism | 2.0 % | 7 |
| Replication, recombination and repair | 2.0 % | 7 |
| Translation, ribosomal structure and biogenesis | 2.0 % | 7 |
| Nucleotide transport and metabolism | 1.7 % | 6 |
| Secondary metabolite biosynthesis, transport and catabolism | 1.2 % | 4 |
| Cell motility | 0.6 % | 2 |
