# Supplementary material for: Molecular markers to characterize the hermaphroditic reproductive system of the planarian Schmidtea mediterranea
Source: BMC Dev Biol. 2011 Nov 10;11:69. doi: 10.1186/1471-213X-11-69 (PMC3224759; doi:10.1186/1471-213X-11-69)
Supplement: Additional file 2 — Table S2 - Clusters of Orthologous Groups (COG) functional categories for genes upregulated in asexual planarians. Genes were assigned putative functions based on their conserved domains. Some genes are assigned more than one functional category. [file 1471-213X-11-69-S2.DOC]

| **COG Functional Category** | **Number of genes** |
| --- | --- |
| Posttranslational modification, protein turnover, chaperones | 3 |
| Signal transduction mechanisms | 3 |
| Function unknown | 2 |
| Carbohydrate transport and metabolism | 1 |
| Transcription | 1 |
| RNA processing and modification | 1 |
| Replication, recombination and repair | 1 |
| Nucleotide transport and metabolism | 1 |
| Coenzyme transport and metabolism | 1 |
| General function prediction only | 1 |
| Cytoskeleton | 1 |
