# Supplementary figures and images for: Molecular markers to characterize the hermaphroditic reproductive system of the planarian Schmidtea mediterranea
Source: BMC Dev Biol. 2011 Nov 10;11:69. doi: 10.1186/1471-213X-11-69 (PMC3224759; doi:10.1186/1471-213X-11-69)

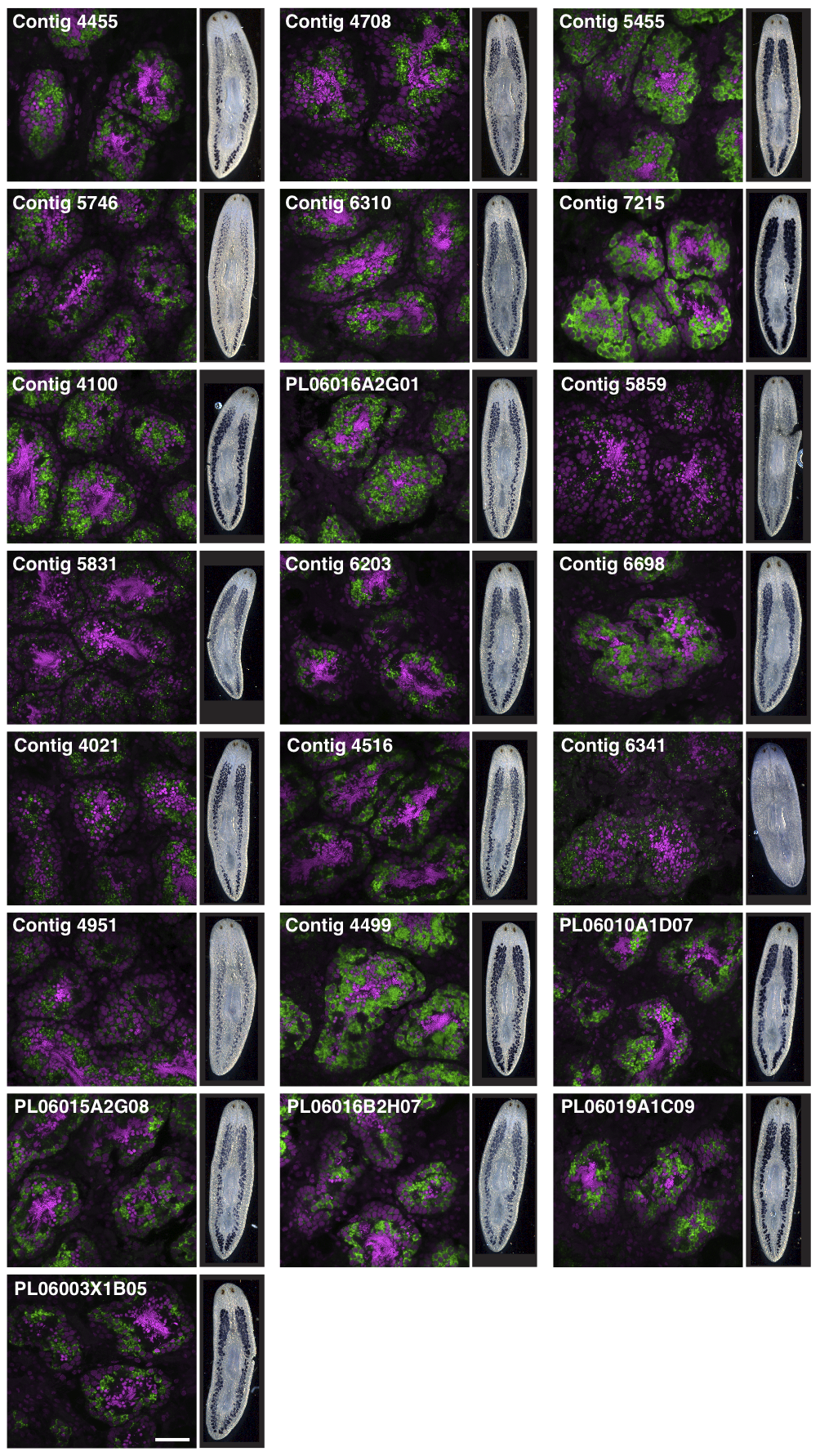

Supplement: Additional file 3 — Figure S1 - Genes upregulated in the sexual planarian that are expressed in the testes. Right panels are fluorescent in situ hybridizations (green), nuclei are counterstained with DAPI (magenta). Left panels are whole mount in situ hybridizations developed with nitro blue tetrazolium/5-bromo-4-chloro-3-inodlyl phosphate (NBT/BCIP). Scale bars: 40 μm. [file 1471-213X-11-69-S3.TIFF]

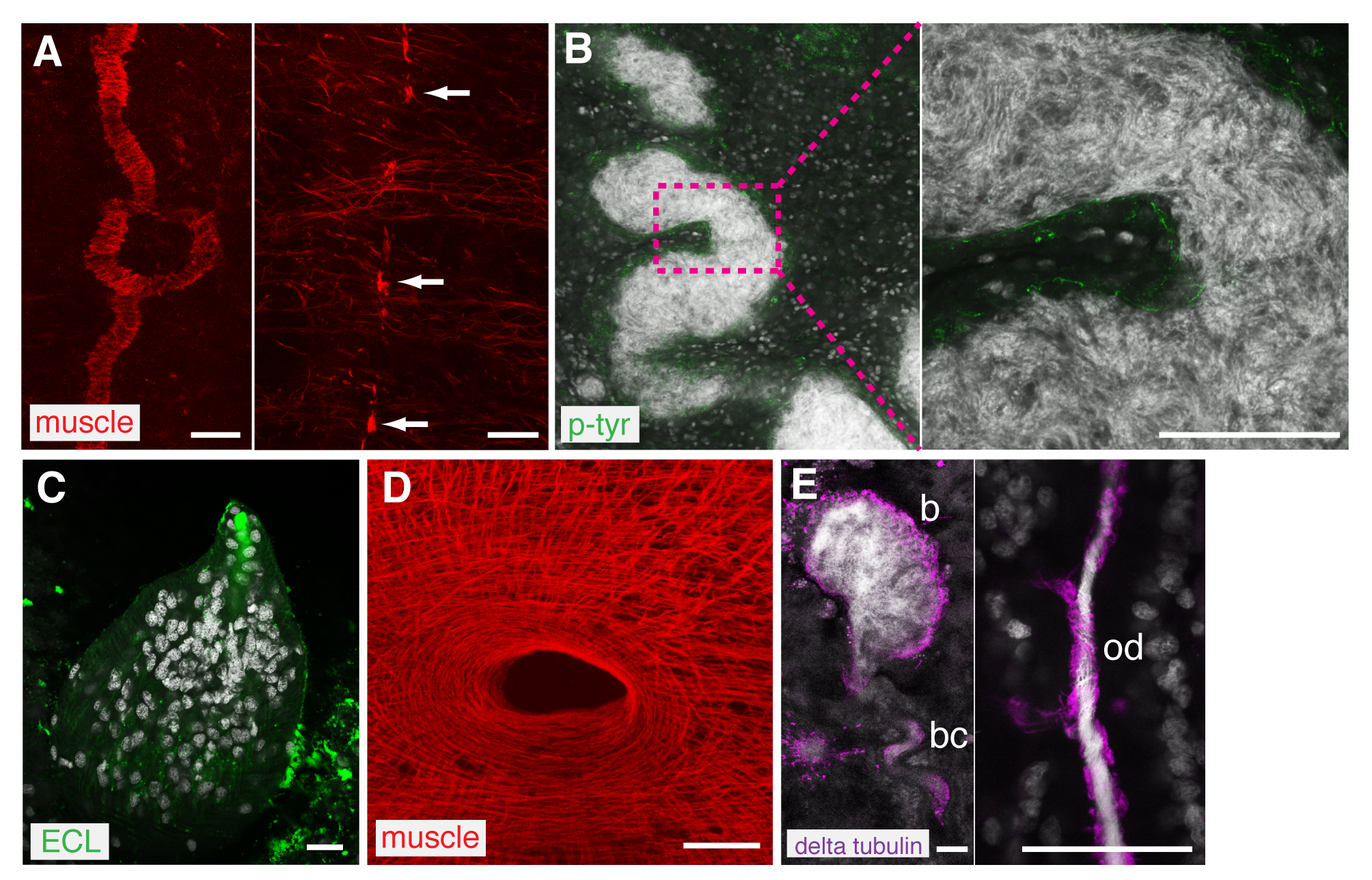

Supplement: Additional file 5 — Figure S2 - Immunofluorescent and lectin labeling of the planarian reproductive system. (A, D, E) Maximum projection of a confocal z-stack. (B, C) Single confocal optical sections. (A, B, D, E) Immunostaining in red/magenta/green, DAPI in grey. (C) Lectin staining in green, DAPI in grey. (A) Looped section of the oviduct, labeled by anti-muscle. Arrows in right image indicate individual oviduct fragments in a sexually immature animal, labeled by anti-muscle. (B) Antibodies against phosphotyrosine (p-tyr) label the seminal vesicles. (C) Erythrina cristagalli lectin (ECL) labels the penis papilla. (D) Muscle fibers of the gonopore are labeled with anti-muscle. (E) Flagella of the sperm are labeled with anti-tubulin δ2 (delta tubulin). Left image shows sperm in the bursa (b) and bursal canal (bc). Right image shows sperm in the oviducts (od). Scale bars: (A, B, D, E) 50 μm; (C) 20 μm. [file 1471-213X-11-69-S5.TIFF]
