# Supplementary material for: Molecular markers to characterize the hermaphroditic reproductive system of the planarian Schmidtea mediterranea
Source: BMC Dev Biol. 2011 Nov 10;11:69. doi: 10.1186/1471-213X-11-69 (PMC3224759; doi:10.1186/1471-213X-11-69)
Supplement: Additional file 4 — Table S3 - Genes upregulated in the sexual planarian that are expressed in the testes and accessory reproductive organs of the planarian. [file 1471-213X-11-69-S4.DOC]

| **ID** | **Homologs in other organisms based on TBLASTX with NR** | **Conserved domain based on Interproscan** | **COG term** | **Gene name referenced in paper** |
| --- | --- | --- | --- | --- |
| PL08003A2D08 / PL06009A1F04 (Contig 4455)  [GenBank: HO005518.1, DN311062.1] | *Schistosoma mansoni* expressed protein (XM_002572512.1) (3e-13); *Schistosoma japonicum* protein (AY814667.1) (3e-11) | Hmg-box | transcription |  |
| PL08003B2B11 / PL06010B2E07 (Contig 4708)  [GenBank: HO005637.1, DN311687.1] | *Paramecium tetraurelia* hypothetical protein (XM_001461995.1) (1e-03) |  | transcription |  |
| PL08004B2A05 / PL06012A1D09 (Contig 5455)  [GenBank: BK007100] | *Dugesia etrusca* Y1 protein (AJ439094.1) (2e-65); *Homo sapiens* CSDA protein variant protein (AB209896.1) (1e-30); *Mus musculus* Y-box binding protein (L35549.1) (1e-30) | Cold shock protein | translation | *Smed-msy4* |
| PL08007B1A06 / PL06018B1A11 (Contig 5746)  [GenBank: HO006595.1, DN314502.1] | *Tribolium castaneum* cAMP-dependent protein kinase R2 (XM_967342.2 ) (3e-64); *Drosophila melanogaster* cAMP-dependent protein kinase R2 (NM_165723.3 ) (1e-62); *Homo sapiens* protein kinase, cAMP-dependent, regulatory, type II alpha (AB464095.1) (9e-59); *Mus musculus* protein kinase, cAMP dependent regulatory, type II alpha (AK132824.1) (1e-58) | Cyclic nucleotide-binding domain cNMP-(binding) | transcription |  |
| PL08009A2A04 / PL06022B2C08 (Contig 6310)  [GenBank: HO007035.1, DN316100.1] | *Drosophila melanogaster* cAMP-dependent protein kinase R2 (NM_165723.3) (2e-88); *Schistosoma mansoni* cAMP-dependent protein kinase type II-alpha regulatory subunit, putative mRNA (XM_002569764.1) (2e-87); *Homo sapiens* protein kinase, cAMP-dependent regulatory, type II, alpha (NM_004157.2) (6e-86) | Cyclic nucleotide-binding domain cNMP-(binding) | transcription | *Smed-pka* |
| PL05015A1B12 (Contig 7215)  [GenBank: DN307831.1] | *Saccoglossus kowalevskii* dorsal switch-like hmg protein (GU211185.1) (0.016) | Hmg-box | transcription | *Smed-thmg-1* |
| PL08002A1G11 / PL06006A1A04 (Contig 4100)  [GenBank: HO005219.1, DN309904.1] | *Danio rerio* protein phosphatase 1, catalytic subunit, alpha isoform b (NM_212710.2) (1e-153); *Xenopus laevis* protein phosphatase 1, catalytic subunit, beta isozyme (NM_001091957.1) (1e-152); *Mus musculus* protein phosphatase 1, catalytic subunit, beta isoform (NM_172707.3) (3e-152); *Homo sapiens* protein phosphatase 1, catalytic subunit, beta isozyme (NM_206876.1) (3e-152) | Metallo-dependent phosphatase | signal transduction |  |
| PL08006B2E03 / PL06016A2G01  [GenBank: HO008078.1, DN313728.1] | *Danio rerio* phosphodiesterase 7A-like (NM_001168263.1) (6e-43); *Homo sapiens* phosphodiesterase 7A (BC058025.1) (7e-43); *Mus musculus* cAMP phosphodiesterase (AY007702.1) (9e-43); *Xenopus tropicalis* phosphodiesterase 7B (BC135159.1) (5e-40) | 3'5'-cyclic nucleotide phosphodiester-ase, catalytic domain | signal transduction | *Smed-pde* |
| PL08008A1A06 / PL06019A2C02 (Contig5859)  [GenBank: HO006734.1, DN314799.1] | *Xenopus laevis* glucose-6-phosphatase (NM_001094213.1) (2e-25); *Mus musculus* glucose 6 phosphatase, catalytic, 3 (NM_175935.3) (3e-25); *Homo sapiens* glucose 6 phosphatase, catalytic, 3 (NM_138387.3) (1e-24); *Mus musculus* glucose-6-phosphatase (BC013448.1)( 6e-20) | Phosphatidic acid phosphatase type 2/haloperoxi-dase (PAP2) | signal transduction |  |
| PL08008A1C09 / PL06019A1D08 (Contig 5831)  [GenBank: HO006752.1, DN314724.1] | *Arabidopsis thaliana* TOPP2; protein serine/threonine phosphatase (NM_125306.2) (2e-68); *Plasmodium vivax* SaI-1 ser/thr protein phosphatase (XM_001616759.1) (3e-67) | Metallo-dependent phosphatase / serine/threonine protein phosphatase | signal transduction |  |
| PL08009A1B07 / PL06021B2G05 (Contig 6203)  [GenBank: HO006984.1, DN315773.1] | *Danio rerio* protein phosphatase 2, regulatory subunit B', delta (NM_213318.1) (9e-113); *Homo sapiens* protein phosphatase 2, regulatory subunit B (BC003593.1) (1e-109); *Mus musculus* protein phosphatase 2, regulatory subunit B (NM_001081457.1) (1e-109); *Xenopus laevis* protein phosphatase 2, regulatory subunit B', gamma (NM_001094169.1) (2e-109) | Protein phosphatase 2A, regulatory B subunit | signal transduction | *Smed-pp2* |
| PL05009A1H08 (Contig 6698)  [GenBank: DN305672.1] | *Danio rerio* thioredoxin domain containing 9 (BC165424.1) (4e-55); *Homo sapiens* ATP binding protein associated with cell differentiation (AK222616.1) (4e-53); *Xenopus laevis* thioredoxin domain containing 9 (NM_001087441.1) (2e-52); *Mus musculus* thioredoxin domain containing 9 (NM_172054.4) (1e-51) | Thioredoxin fold / thioredoxin-like fold | signal transduction |  |
| PL08002A1B11 / PL06005A2G06 (Contig 4021)  [GenBank: HO005179.1, DN309696.1] | *Schistosoma mansoni* carbonic anhydrase II (XM_002574262.1) (3e-36); *Homo sapiens* carbonic anhydrase I (DQ894091.2) (3e-27); *Mus musculus* carbonic anhydrase-like (AK004896.1) (1e-26); *Danio rerio* carbonic anhydrase VII (BC154317.1) (7e-26) | Carbonic anhydrase | function unknown |  |
| PL08003A2C08 / PL06009B1E05 (Contig 4516)  [GenBank: HO005509.1, DN311232.1] | *Trichoplax adhaerens* hypothetical protein (XM_002111697.1) (4e-11), *Homo sapiens* kinesin light chain 1 (AB451431.1)( 2e-08); *Caenorhabditis elegans* Kinesin Light Chain family member (klc-2) (NM_001028493.2) (3e-08) | Tetratrico-peptide-like helical | function unknown | *Smed-tplh* |
| PL08003B2A11 / PL06010A1E04 (Contig 6341)  [GenBank: HO005628.1, DN311414.1] |  |  | function unknown |  |
| PL08004B1H12 / PL06012B2C11 (Contig 4951)  [GenBank: HO005874.1,  DN312402.1] | *Gallus gallus* tetraspanin 15 (NM_001006499.1) (8e-05); *Homo sapiens* tetraspanin 15 (NM_012339.3) (1e-03); *Mus musculus* tetraspanin 15 (NM_197996.2) (3e-03) | Tetraspanin, EC2 domain | function unknown |  |
| PL05005A1F08 (Contig 5255)  [GenBank: DN304193.1] | *Bos taurus* granulin (BT026179.1) (7e-23); *Xenopus laevis* granulin (NM_001087209.1) (6e-21); *Homo sapiens* granulin (BT006844.1) (1e-20); *Danio rerio* granulin a (NM_001001949.2) (3e-20) | Granulin | function unknown | *Smed-grn* |
| PL05007B1D03 / (Contig 874)  [GenBank: DN305069.1] | *Strongylocentrotus purpuratus* similar to tetraspanin family protein (XM_001175698.1) (0.13) | Tetraspanin, EC2 domain | function unknown | *Smed-tsp-1* |
| PL08003A1B11 / PL06009B1A11 (Contig 4499)  [GenBank: HO007660.1,  DN311193.1] | *Homo sapiens* plastin 3 (NM_005032.5) (3e-41); *Mus musculus* plastin 3 (NM_145629.2) (3e-41); *Xenopus laevis* plastin 3 (NM_001090112.1) ( 4e-40) | Actinin-type, actin-binding, conserved site,  Calponin homology domain | cytoskeleton | *Smed-plastin* |
| PL08003B1H02 / PL06010A1D07  [GenBank: HO007734.1,  DN311405.1] | *Mus musculus* lymphocyte cytosolic protein 1 (BC022943.1) (2e-41); *Homo sapiens* lymphocyte cytosolic protein 1 (L-plastin) (NM_002298.4) (5e-41); *Schistosoma mansoni* fimbrin (XM_002575031.1) (1e-40) | Actinin-type, actin-binding, conserved site,  Calponin homology domain | cytoskeleton |  |
| PL08006A2G09 / PL06015A2G08  [GenBank: HO008033.1,  DN313360.1] | *Xenopus (Silurana) tropicalis* kinesin-4-like (XM_002932825.1) (1e-29); *Strongylocentrotus purpuratus* similar to C-terminal kinesin (XM_001194846.1) (4e-28) | Kinesin, motor domain | cytoskeleton |  |
| PL08007A1A01 / PL06016B2H07  [GenBank: HO008093.1,  DN313931.1] | *Xenopus tropicalis* tektin 3 (BC135257.1) (8e-46); *Xenopus laevis* tektin 3 (NM_001086388.1) (4e-44); *Mus musculus* tektin 3 (NM_027660.1) (2e-39); *Danio rerio* tektin 3 (XM_696077.2) (5e-39); *Homo sapiens* tektin 3 (NM_031898.2) (3e-38) | Tektin | cytoskeleton | *Smed-tkn-2* |
| PL08008A1B01 / PL06019A1C09  [GenBank: HO008203.1,  DN314713.1] | *Mus musculus* kinesin family member 5A (BC027115.1) (4e-92); *Danio rerio* kinesin family member 5C (NM_001123275.1) (4e-92); *Homo sapiens* kinesin family member 5A (BC150208.1) ( 9e-92); *Schistosoma mansoni* kinesin heavy chain (XM_002571530.1) (5e-91) | Kinesin, motor domain | cytoskeleton |  |
| PL08010A1B05 / PL06003X1B05 (Contig 3817)  [GenBank: HO007229.1,  DN309082.1] | *Ciona intestinalis* similar to tektin 1 (XM_002130430.1) (5e-78); *Xenopus laevis* tektin 1 (NM_001091757.1) (1e-70); *Homo sapiens* tektin 1 (NM_053285.1) (6e-69); *Mus musculus* tektin 1 (NM_011569.2) (4e-67) | Tektin | cytoskeleton | *Smed-tkn-1* |
